# Co-production in the Lost Mothers Project: transforming criminal justice narratives through Lived experience engagement

**DOI:** 10.1186/s40900-024-00583-1

**Published:** 2024-06-05

**Authors:** Laura Abbott, Kate Chivers, Tuesdae Moncrieffe

**Affiliations:** 1 University of Hertfordshire, Hatfield, Hertfordshire UK; 2 Head of Engagement, Birth Companions, London, UK; 3 Lived Experience Team, Birth Companions and Lost Mothers Project, London, UK

**Keywords:** Lived experience, Lost Mothers, Co-production, Collaboration, Criminal justice system

## Abstract

The Lost Mothers Project researches the repercussions of mandatory separation between newborns and women in the Criminal Justice System (CJS), aiming to address gaps in evidence and decision-making for pregnant women within the CJS. Co-production with Birth Companions and their Lived Experience Team (LET) is integral, involving substantial input from the LET in various aspects. This paper, written collaboratively, explores the success stories, challenges, and impact of co-production on The Lost Mothers Project, emphasising the profound role of the LET in reshaping the criminal justice landscape for mothers within the system.

The LET, comprised of mothers with direct CJS experience, assumes a central role as co-creators and decision-makers, providing invaluable insights into systemic issues. The co-design process, exemplified by refining the project's title and logo, showcases collaborative efforts to reduce isolation and emphasise the transformative power of co-production. Challenges in co-production, such as power dynamics and language barriers, are acknowledged, with strategies for overcoming them discussed. The project's commitment to non-hierarchical co-production ensures equal partnership among all stakeholders. Remuneration for the LET is prioritised, avoiding tokenistic engagement.

The co-production paradigm in The Lost Mothers Project contributes to a more compassionate, equitable, and effective criminal justice system. This article concludes that co-production is not just a slogan but a cornerstone for empowering sometimes disempowered populations and fostering positive change in the criminal justice landscape. The transformative impact of the LET in actively shaping the research, coupled with their role as decision-makers, highlights the significance of lived experience engagement in reshaping narratives and creating inclusive research practices within criminal justice studies.

## Abstract (plain language summary)

The Lost Mothers Project looks into how separating newborns from mothers in the Criminal Justice System affects women and staff. The research is trying to fill in the missing information and improve how decisions are made. This research, in partnership with Birth Companions and their Lived Experience Team (LET), focuses on getting direct input from mothers who have been in the justice system.

The LET, made up of mothers with their own experiences of the system, is crucial in shaping the research. They actively contribute to decisions, like refining the project's title and logo. This involvement aims to make sure the research is not just about them but includes their perspectives, reducing feelings of isolation.

Challenges in this collaborative process, such as power dynamics and language barriers, are recognised and strategies to overcome them are discussed. The project commits to a fair and equal partnership among everyone involved, and the LET is compensated for their time.

The co-production approach in The Lost Mothers Project is seen as a way to create more understanding, and equality when undertaking research. The article stresses that co-production is not just a trendy idea but a crucial part of making positive changes in how we understand and address issues in the criminal justice system. The LET's active role, both in shaping the research and making decisions, shows the real impact of always including the viewpoints of people who have personally been through the prison system.

This collaboratively written article is interwoven with quotations from members of the LET, utilising pseudonyms in certain instances. The paper was initially discussed with the team at one of our regular meetings, where volunteers were invited to contribute; consent was always sought for quotes and contributions. Iterations of the paper have been exchanged back and forth, ensuring accuracy, and relevant papers used as references were collectively read, reviewed and agreed upon.

## Background

The Lost Mothers Study investigates the repercussions of mandatory separation between newborn babies and women involved with the Criminal Justice System (CJS). The research seeks to address existing gaps in both the available evidence and the decision-making processes concerning pregnant women within the CJS. The significance of this study arises from the considerable risk that compulsory separations pose to the mental well-being of these women [1]. It builds upon prior research related to the experiences of imprisoned pregnant women [2] and contributes original and robust evidence for initiatives aimed at improving support, information, and the overall experiences of women within health, criminal justice, and social care services.

Co-production, in collaboration with the charity Birth Companions (https://www.birthcompanions.org.uk/) and members of their LET, has been an integral and consistent component of the research from its inception. Every facet of the study, ranging from the formulation of interview schedules, collaborative writing endeavours, to the design of our project logo, incorporates substantial input from the LET. This engagement extends to the scrutiny of language and deliberations on emergent issues. To facilitate this ongoing collaboration, we conduct regular meetings, a blend of online and face-to-face workshops, with all participants being duly compensated for their time and travel. The project also benefits from the insights of an advisory board comprising women with lived experience, including members of the lost mother’s project LET, health, prison and social work professionals, academics, third sector organisations, and a Member of Parliament.

Our approach is marked by a willingness to pose challenging questions and, when necessary, to adapt our research direction accordingly. Our overarching objective is to foster a meaningful impact through our work, recognising co-production as a fundamental and continuous element woven into the fabric of our research. This is not merely an adjunct or a buzzword but a substantive and sustained commitment to collaborative research that transcends traditional boundaries. An essential consideration, particularly when collaborating with women who may have undergone trauma, lies in the nature of our engagement efforts and the manner in which we approach it with sensitivity. Although we may not explicitly label it as such, there seems to be a beneficial shift towards characterising this approach as 'trauma-informed engagement.' Emphasising a trauma-informed approach across all facets of our work is crucial in ensuring its significance. The trauma-informed approach model is grounded in Stephanie Covington's framework [3], focusing on recognising and addressing potential ‘triggers’ that may evoke past traumas in women, thereby enabling us to be trauma-responsive. For instance, all members of the LET and indeed, all members of the Lost Mothers Team are provided with opportunities to debrief after meetings or events to address any hidden triggers. These debriefing sessions are typically facilitated by a staff member from Birth Companions:

*“Birth Companions offer trauma-informed, women led, non- judgemental support during the critical 1001 days of pregnancy and early motherhood. Our engagement activities focus on increasing understanding and creating positive change within the systems women are impacted by. This work is underpinned by the key principles of a trauma informed approach, a thread which runs through everything that we do. Trauma-informed engagement ensures that women feel empowered to share their experiences in a safe, supportive space.”* Kate Chivers – Head of Engagement

## Main text

### Introduction

In the quest for a more compassionate and equitable criminal justice system, the power of co-production has emerged as a guiding principle in the transformative journey of The Lost Mothers Project. This project, at its heart, is a testament to the invaluable role that lived experience teams play in reshaping the landscape of criminal justice, specifically concerning mothers within the system. In this article, we delve into the success stories, challenges, and the profound impact of co-production on The Lost Mothers Project, highlighting the significance of partnership with community organisations, charities, policymakers, and prisons.

### The role of lived experience teams

*As a member of the lived experience team it has been amazing to be included in the entire research process, especially as someone who contributes a lot to projects. I feel like I’ve got the luxury from being here from the beginning*. (Suzy)

At the core of The Lost Mothers Project is the concept of co-production. Co-production, as the guiding principle, emphasises meaningful engagement and active involvement of stakeholders in all stages of the project. As professionals, as members of LET, experts and academics, we all see things from different perspectives. In our approach, we emphasise how lived experience individuals and charities are not just participants but co-creators and decision-makers, ensuring that the project genuinely represents diverse perspectives. Kaisler and Missbach's [4] guide on co-creation for researchers underscores the critical significance of involving experts by experience in the research process from its inception. It emphasises that establishing a foundation of trust between researchers and patients is a paramount prerequisite. The LET takes centre stage in this co-production paradigm. The team consists of mothers who have first-hand experience with the criminal justice system and prison:

*All of my input feels valuable and not just for the project itself, but for my own personal well-being. It has been amazing so far. You often feel like after you’ve done something that you are capable of anything. It puts a battery in your back and makes you feel more powerful, I feel more purposeful.* (Tuesdae)

This unique experience offers unparalleled insights into the systemic issues that perpetuate cycles of incarceration. By embracing this expertise, we collaboratively design the project that is more attuned to the distinct needs and realities of an often-marginalised population. The power of the LET voices and the authenticity of their insights are instrumental in driving the project forward. In Fox’s 2020 paper [5] it is acknowledged that the experts by experience acknowledged a presence of a cohesive team dynamic, which they deemed instrumental in facilitating positive engagement experiences. Bandola-Gill et al. (2023)’s systematic review found that Co-production is increasingly recognised as both a theoretical concept and a practical strategy, yet its definition varies across disciplines and settings [6]. They identified five separate meanings of co-production: as a science-politics relationship; knowledge democracy; transdisciplinary; boundary management; and evidence-use intervention. Their discussions highlighted conflicts between different concepts of co-production, particularly between knowledge consensus and evidence-use intervention. They emphasise the importance of interdisciplinary collaboration and clarity in defining co-production for researchers and practitioners. Furthermore, they suggest that funders should prioritise the processes involved with co-production rather than just the outcomes.

## Methodology

The intended outputs of the Lost Mothers Project include providing insights into the experiences of health and social care professionals and prison officers involved in the care of incarcerated women separated from their newborns. The findings will be utilised to inform future policy and support initiatives in this domain, aiming to enhance the care and support provided to mothers. Data collection includes qualitative interviews and observations of decision-making boards by the Principal Investigator and Research Assistants. Our recruitment strategy of LET members involved early project presentations, inviting interested individuals to apply through Birth Companions initially as expressions of interest. The inaugural workshop took place in London before the project commenced and included six members of the LET who had expressed interest. Careful planning ensured that all subsequent workshops accommodate childcare needs, with provision expenses covered if necessary. The agenda is collaboratively developed at least three weeks in advance, featuring structured discussion points; for instance, one meeting addressed the concept of "non-engagement" with health professionals, a topic that had emerged from qualitative interviews with health professionals. Icebreakers, such ‘if you could choose a superpower, what would it be’ start our sessions, fostering a relaxed and informal atmosphere. Face-to-face meetings include refreshments and regular breaks to facilitate engagement and comfort. Our meetings take place every two months. blending both online and in-person formats. Online sessions span two hours, while face-to-face workshops, including lunch, extend to four hours. The Birth Companions team typically arranges venues for in-person gatherings, often selecting locations in London to ensure accessibility. Apart from the LET meetings, we convene an advisory board comprising academics, health and social care professionals, and a politician, gathering quarterly. Two members of the LET team regularly participate in these sessions to ensure representation of their perspectives.

The first meeting took place at a central location, ensuring accessibility for all attendees, including members of the research team, a representative from Birth Companions, and the LET for the Lost Mothers project. This meeting was marked by a candid exchange of insights. Attendees introduced themselves, sharing their connections to Birth Companions and explaining the organisation's significance in their lives. Subsequently, the research team presented the Lost Mothers project, emphasising the vital role that the LET would play in owning the project. This first co-design workshop, included refining the project's title, designing a logo, and ensuring project language aligned with the LET's experiences. The pre-existing partnership between Lost Mothers and Birth Companions, established with legal backing from the University of Hertfordshire, ensured LET's sustained support and recognition. This and subsequent workshops effectively exemplify co-production, integrating the LET's perspectives into the project:

*We were getting to hear how people think and their thought processes, so I feel like we gelled quite well because we all see things from a different lens which I think has made our research stronger. Personally, it has made me feel like an integral member of the team and not just an accessory or a tick boxing exercise as often when I am engaging with other projects, it kind of feels like that.* (Tuesdae)

### Participation in Refining the Project's Title and logo – co-production in action

Initiating engagement with all stakeholders at the project's outset laid the groundwork for our research endeavours. The Lost Mother's Project is regarded as a collective effort, fostering inclusivity and a sense of ownership. During our initial discussions in the first workshop, the phrase "feeling lost" emerged, prompting a comprehensive exploration of the project's title. The attendees collectively assessed whether the term "lost mothers" aptly encapsulated the project's overarching theme. The group reached a consensus that "lost" indeed accurately described the emotional spectrum associated with their experiences of being mothers in prison, symbolising isolation, undeserved circumstances, and a sense of being adrift in a forgotten and empty space. Women talked more of how they connected with the word lost and that to them it symbolised:

*Feeling out at sea; alone; isolated; undeserved; caught in the middle; feeling forgotten about; in a lost and empty space and losing who you are*. (LET)

Furthermore, the meeting was dedicated to co-designing a logo for the Lost Mothers project. To facilitate this creative process, the research team procured art supplies. One of the women took a lead role in this endeavour, crafting a visually striking logo from pink/mauve crepe paper in a relatively short timeframe:

*“The heart within a heart symbolises two lives, the big heart the mother and the little heart, the womb or the child. The birds flying out symbolises the mothers broken heart with pieces floating away, the relevance of the number three representing pregnancy, birth and postnatal.”* (LET)

**Fig. 1:**
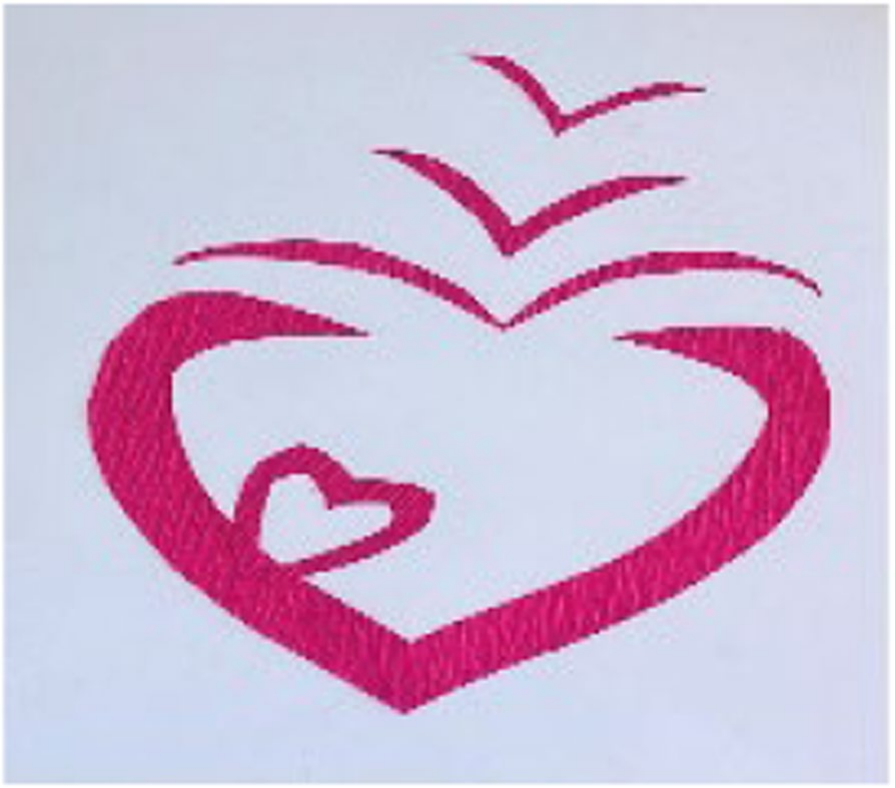
Logo designed by the LET for the Lost Mother's Project

The logo was subsequently adapted by a graphic designer and agreed by the LET for use on our website and materials Fig. 1.

This meeting underscored the significance of involving the LET in co-design activities and emphasised their pivotal role in the project's ultimate success, prior to data collection and ensuring inclusion from the start.

### Connectedness and reducing isolation

Connectedness is a pivotal force in the co-production process [7]. Through meaningful engagement with the lived experience team and the charity Birth Companions, the Lost Mothers Project hopes to reduce the sense of isolation and marginalisation that often accompanies experiences within the criminal justice system. Inclusivity is a common goal in co-producing research [8, 9]. In collaboration with the LET, the project becomes a collective endeavour, where every team member has a sense of agency and ownership. Their involvement is not passive; it is transformative:

*Using co-production with the lived experience team tends to be more powerful than estimated not only for the validity in the work being produced, but for the women as directly being included in the process. This can positively impact on their wellbeing, feeling like their input is a catalyst for change. It didn’t feel like a tick boxing exercises like we have experienced in the past*. (Kate, Head of Engagement at Birth Companions)

The transformative worth of co-production, particularly under the leadership of mothers with lived experience, transcends conventional project development models. Its significance extends beyond the mere validation of the work produced, resonating with an impact on the well-being of the women involved. The empowerment derived from active participation in decision-making processes serves as a potent catalyst for change. This underscores the notion that co-production is not a mere conceptual framework but a substantiated instrument for catalysing meaningful change in a manner distinct from past engagements which have sometimes felt like tick box exercises to the LET:

*I find that sometimes researchers have a certain expectation from us, they will want to hear what we have to say for a short period of time, but often you don’t have any involvement, or any say, so the research is shaped more from one perspective other than our lived experience*. (Suzy)

### Challenges faced and strategies to overcome

In the course of co-production, we remain cognisant of the challenges that may manifest [10]. Power dynamics, language barriers, stigma, and resource limitations can be formidable obstacles. However, these challenges are not insurmountable. The Lost Mothers Project, in its commitment to co-production, actively addresses these issues by collaborating with community and charity organisations, policymakers, and prisons. Johnson et al. (2016) [11] explain that meaningful engagement of ‘patients’ in research endeavours represents a substantial commitment of time and resources where adaptability is key, and the cultivation of mutual trust and the demonstration of unwavering integrity are essential:

*I would like to add I would say I would say some of the challenges that I faced are sometimes that there are technical words and things* (e.g. medical terms and laws) *we don’t understand but we are so blessed to have a team of experts that will take the time to explain to you and we have notebook so if there’s something I don’t understand I’ll take notes. I’ll wait until the end* (of meetings / workshops) *so I can go and read up and educate myself. This has been such enriched activity because I have learnt so much*. (Suzy)

By forging strategic partnerships, we navigate the intricate web of challenges, ensuring that the co-production process remains inclusive, impactful, and effective. We understand that co-production is not a solitary endeavour but a collective effort to overcome barriers and create a more compassionate and equitable criminal justice system. Throughout, we encountered various challenges, including logistical hurdles, differing stakeholder priorities, and resource limitations. To address these obstacles, we prioritised open communication and fostered a culture of mutual respect and understanding. Regular dialogue and feedback sessions allowed us to identify common goals and areas of divergence, facilitating alignment and consensus-building:

*I would say timing and scheduling has been a barrier. The language sometimes used can be a barrier, but Laura and Kate were always on hand to expound. We were always provided with sufficient materials and references to keep ourselves informed if we chose to.* (Tuesdae)

In one of our workshops, we talked about the phrase "criminalised women," which had been used on our Lost Mothers website. The women who had been through the justice system did not like the label. They felt it was unfair, making it sound like they can never move past their past mistakes and grow. It sparked a thought-provoking conversation for all of us, making us reconsider the impact of labels like these in our discussions.

### A collective pursuit

Williams et al. (2020) [10] assert that active engagement in projects goes beyond mere participation and that the term co-production is sometimes misconstrued in academic research contexts. They contend that to rectify imbalances of power, a critical emphasis should be placed on co-production. Considering this perspective, the Lost Mothers project strives to ensure that its co-production endeavours are characterised by inclusivity with a distinctly non-hierarchical approach. This commitment to non-hierarchical co-production was woven into the fabric of the Lost Mothers project from the beginning. The project operates on the fundamental principle that all stakeholders, ranging from research assistants to advisory boards to the entire project team, stand as equal partners in the collaborative enterprise (Fig. 3).

### The importance of renumeration—moving beyond the biscuit

Since the initiation of The Lost Mothers' project, we prioritised compensating the time of our LET members. While academics, researchers, and charity staff receive remuneration for their time, it is common for individuals whose contributions to research are deemed equal to go unrewarded or receive compensation in the form of vouchers. Providing an honorarium for their time is not only crucial but also serves as a signal that the value of the LET is considered equal rather than an ancillary component. There have been instances where organisations may engage with those who have lived experience, delving into traumatic experiences, which can feel akin to exploitation as explained by a LET member:

*Many times organisations engage with us wanting to repeatedly go over our trauma feeling like we are almost being exploited whilst everyone else in attendance is being paid*. (Natasha)

Perry and Mullins (2023) [12], in collaboration with the National Institute for Health Research (NIHR), question the traditional approach of "£25 and a biscuit" in public engagement. They propose a shift away from mere tokenistic involvement, where checkboxes are ticked for funding, towards a transformative paradigm. A member of a third sector organisation offered feedback on the Perry and Mullin’s (2023) paper:

*The article definitely chimes with our experiences as an organisation, particularly the paucity of research to show what good engagement looks like. Also, the formulaic nature of the approach to engagement and a lack of understanding about the barriers women face and flexibility needed to enable women to take part in activities. When I have initial conversations with researchers, I have to work out if they are fully flexible or if they just want to get the engagement completed as quickly and as easily as possible. The emails with ‘I need to speak to some women in the next 2 weeks about….’ still keep coming, short time frames, not understanding the time and care needed to carry out engagement. We need deeper listening, moving beyond the biscuit, as the article said, to get a seat at a different table where we write the menu*. (Third sector organisation member)

**Fig. 2:**
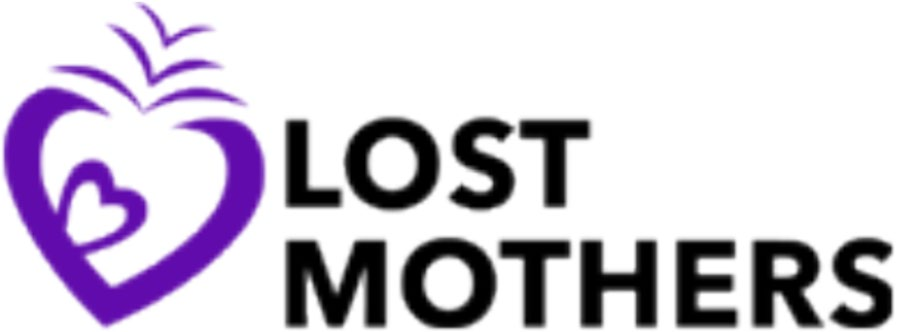
Final graphic designed logo adapted from original and agreed by team

**Fig. 3:**
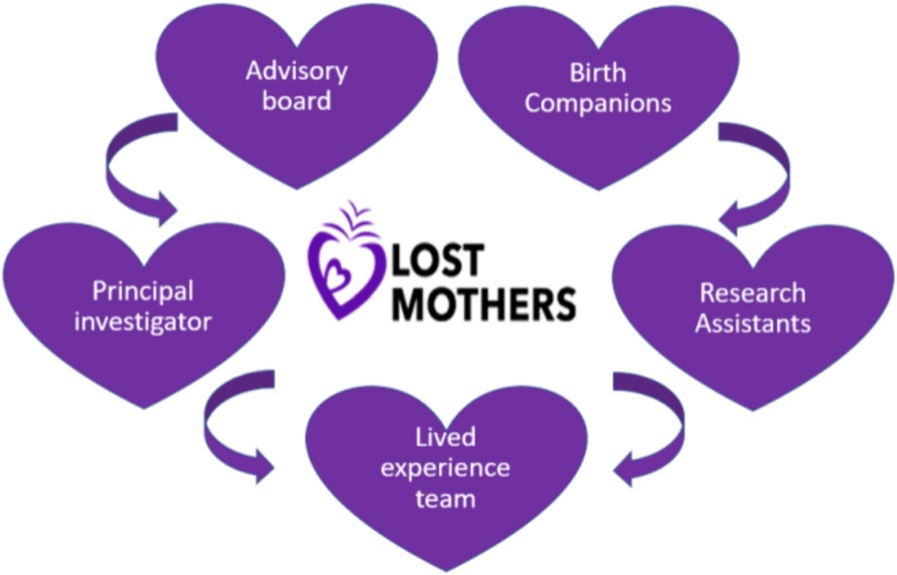
Circle of Co-production

This new perspective advocates for a revolution in public engagement, emphasising the need for frameworks that genuinely prioritise and elevate engagement to a distinct and meaningful position (Figs. 2 and 3).

## Discussion

The founding principle of The Lost Mothers Project lies in the overarching concept of co-production. Co-production, serving as the project's guiding ethos, underscores the imperative of substantive engagement and active participation of stakeholders across all phases of the project. Our approach is fundamentally centred on the acknowledgment that individuals with lived experiences and charitable organisations are not mere participants but, in fact, co-creators and decision-makers, thus ensuring the genuine representation of a wide array of perspectives.

Michaela Booth (Patient Engagement Lead, Care UK) and Paula Harriott, (Head of Prisoner Engagement at the Prison Reform Trust) are two women with lived prison experience and extensive participation in prison research projects. In their book chapter they shed light on the lived experience of being researched [13].

They voiced apprehensions regarding the possible misuse and absence of consent in the research process, especially in the investigation of prison experiences. This raises concerns about fairness and ethical considerations in research, particularly concerning women who have undergone imprisonment. They underscore this as a potential risk, expressing worry about the likelihood of their collective and individual experiences being utilised, manipulated, and presented without their explicit knowledge or authentic consent:

*Stepping into our identities as research subjects, recalling experiences which have been dominated by expressing our pain, misery and brokenness to fit a preconceived narrative is painful for us, and reduces our duality of strength and fragility to a prewritten script that fits, but does not challenge. Surely all research needs to be ground-breaking, not replication of pre-existing knowledge repackaged to look new. For both of us living as criminalised women in the system, we know that “if we always do what we always did, we will always get what we always got!” Understanding that deeply is what has pushed us forward as we seek both personal and systemic change.* (Booth and Harriott, 2021: 216)

Booth and Harriott's analysis emphasises the ethical obligation to confront power imbalances, clarify research purpose, establish ownership, and uphold overall research conduct. Their contribution offers valuable insights to the wider academic discourse on the ethical intricacies involved in conducting research with marginalised populations.

In alignment with the guidance provided by Kaisler and Missbach in their research co-creation guide, the pivotal importance of incorporating individuals with expertise derived from their own experiences at the outset of the research process is underscored. This emphasises the fundamental prerequisite of cultivating a bedrock of trust between researchers and individuals with lived experiences. The crux of this co-production paradigm is embodied by the Lived Experience Team at The Lost Mothers’ project, the most central element within the project framework. Comprising mothers who possess first-hand knowledge of the criminal justice system and the penal system, the LET assumes this central role in manifesting the principles of co-production.

In their comprehensive systematic review, Greenhalgh [14] and colleagues undertook an investigation into the utilisation of frameworks designed to facilitate co-production within the realm of research. Their analysis revealed a prevailing pattern wherein these frameworks were predominantly employed solely by their creators, thus largely failing to garner broader adoption. Moreover, the papers reviewed uniformly advocated against the perfunctory or superficial involvement of stakeholders, emphasising the imperative to avoid tokenistic engagement in the co-production process.

Through the establishment of strategic partnerships, a deliberate effort is made to navigate the intricate matrix of challenges, thus ensuring that the co-production process remains inclusive, efficacious, and transformative. It is firmly recognised that co-production is not an isolated pursuit, but a collective endeavour aimed at transcending obstacles and fostering a more compassionate and equitable criminal justice system (Table 1).

**Table 1:** Recommendations for Researchers: How to Manage Co-Production Authentically and Inclusively

No.	Recommendation
1.	Engage from the Offset: The project is to be shared, and lived experience is not to be used as an add-on.
2.	Payment: Set up contracts early; handle paperwork and legalities promptly to ensure smooth payment to lived experience members.
3.	Activities: Plan inclusive and enjoyable activities with productive outcomes such as writing retreats.
4.	Permissions and Check-Ins: Revisit consent regularly to ensure ongoing approval. Ensure confidentiality at all times.
5.	Value Proposition: Always consider the benefits for the lived experience team members as a whole.
6.	Child Care Consideration: Account for child care needs, aligning activities with school hours and providing mutually convenient venues.
7.	Accessibility: Ensure venues and activities are accessible to all members, considering mobility and other accessibility requirements.

## Conclusions

The Lost Mothers Project firmly believes that involving mothers with lived experience of criminal justice settings and prison as active co-producers yields transformative benefits for all stakeholders involved. Through empowerment, fostering connectedness, and acknowledging expertise, we contribute to the collective pursuit of a more compassionate, equitable, and effective criminal justice system. This is summed up in a concluding comment:

*I hope from reading this and getting to see the results from our research and all of the rich work that we have done by collaborating as a team that other charities other organisations will mirror this. The one thing I hope that people get from this is that having an uplifting experience, the team can be as fruitful as you want it to be, and that it’s not just for the end, it can be from the beginning and for the middle. It is not just an accessory - this could be pivotal in your research, in your work and in your day-to-day operations.* (Tuesdae)

As we move forward, we remain committed to the idea that co-production is not a mere slogan but a cornerstone in empowering vulnerable populations and fostering positive change within the criminal justice landscape. We look forward to engaging with research teams, exchanging insights, and sharing best practices, as together we work towards a future where co-production becomes the norm, rather than the exception, in redefining and humanising the criminal justice system.

## Data Availability

No datasets were generated or analysed during the current study.
